# Association of Trauma Type, Age of Exposure, and Frequency in Childhood and Adolescence With Psychotic Experiences in Early Adulthood

**DOI:** 10.1001/jamapsychiatry.2018.3155

**Published:** 2018-11-21

**Authors:** Jazz Croft, Jon Heron, Christoph Teufel, Mary Cannon, Dieter Wolke, Andrew Thompson, Lotte Houtepen, Stanley Zammit

**Affiliations:** 1 Centre for Academic Mental Health, Population Health Sciences, Bristol Medical School, University of Bristol, Bristol, United Kingdom; 2 Cardiff University Brain Research Imaging Centre, School of Psychology, University of Cardiff, Cardiff, United Kingdom; 3 Department of Psychiatry, Royal College of Surgeons in Ireland, Dublin, Ireland; 4 Division of Mental Health & Wellbeing, Department of Psychology, University of Warwick, Warwick, United Kingdom; 5 Warwick Medical School, University of Warwick, Warwick, United Kingdom; 6 Medical Research Council Centre for Neuropsychiatric Genetics and Genomics, Division of Psychological Medicine and Clinical Neurosciences, Cardiff University School of Medicine, Cardiff, United Kingdom

## Abstract

**Importance:**

Cross-sectional and longitudinal studies have consistently reported associations between childhood trauma and psychotic experiences and disorders. However, few studies have examined whether the age of exposure or specific trauma types are differently associated with the risk of developing psychotic experiences.

**Objective:**

To examine whether exposure to trauma, assessed at multiple age periods between 0 and 17 years of age, is associated with an increased risk of psychotic experiences by age 18 years and whether this association varies according to trauma type as well as age and frequency of exposure.

**Design, Setting, and Participants:**

This study used data from the Avon Longitudinal Study of Parents and Children, a large population-based birth cohort in the United Kingdom that recruited women who resided in the Avon Health Authority area and had an expected delivery date between April 1, 1991, and December 31, 1992. Data on psychotic experiences were included in the study, along with trauma variables derived from assessments completed by the parents or self-reported by the participants. The variables represent exposure to any trauma type between ages 0 and 17 years; any trauma type within a distinct age period: early childhood (0-4.9 years), middle childhood (5-10.9 years), or adolescence (11-17 years); specific trauma types between ages 0 and 17 years; and specific trauma types within early childhood, middle childhood, or adolescence. Data were analyzed from January 9, 2017, to November 30, 2017.

**Main Outcomes and Measures:**

Suspected or definite psychotic experiences were assessed using the psychosis-like symptoms semistructured interview at age 12 years and then at age 18 years.

**Results:**

The sample of 4433 participants included 2504 (56.5%) females, with a mean (SD) age of 17.8 (0.38) years. Exposure to any trauma up to age 17 years was associated with increased odds of psychotic experiences at age 18 years (adjusted odds ratio, 2.91; 95% CI, 2.15-3.93). All trauma types from age 0 to 17 years were associated with an increased odds of psychotic experiences. The population-attributable fraction for childhood and adolescent trauma on psychotic experiences at age 18 years was 45% (95% CI, 25%-60%). Effect sizes for most trauma types were greater for exposure that was more proximal to the outcome, although CIs overlapped with those for more distal trauma. Evidence supported dose-response associations for exposure to multiple trauma types and at multiple age periods. In an analysis aimed at minimizing reverse causality, adolescent trauma was also associated with past-year incident psychotic experiences at age 18 years.

**Conclusions and Relevance:**

These findings are consistent with the thesis that trauma could have a causal association with psychotic experiences; if so, identification of modifiable mediators is required to inform prevention strategies.

## Key Points

**Question:**

Is exposure to trauma during childhood and adolescence associated with an increase in the risk of developing psychotic experiences?

**Findings:**

In this cohort study of 4433 children and adolescents, all types of trauma, at any time from early childhood through adolescence, are associated with subsequent psychotic experiences after adjusting for plausible confounders. Effect sizes were larger for repeated exposure, exposure to multiple types of trauma, and for more proximal exposure to trauma.

**Meaning:**

These findings are consistent with the thesis that trauma could have a causal association with psychotic experiences; if so, identification of modifiable mediators is required to inform prevention strategies.

## Teaser

This population-based cohort study investigates whether the type, frequency, and severity of trauma encountered during childhood is associated with an increase in the likelihood of hallucinations, delusions, thought interference, and other psychotic experiences in young adulthood.

## Introduction

Meta-analyses show that exposure to childhood trauma is associated with a 2- to 3-fold increase in risk of psychotic outcomes.^1,2,3,4^ Increasing severity or chronicity of trauma plus the presence of multiple different types of trauma exposure (eg, physical and emotional abuse), which frequently co-occur,^5^ further elevates this risk.^6,7,8,9,10^

However, substantial heterogeneity in effect sizes has been observed across studies.^11,12,13^ Methodological issues, such as small sample sizes, cross-sectional data, variation in how trauma and psychotic experiences were assessed, and extent of adjustment for confounding, could explain this heterogeneity. As a result, it is not determined whether the association between trauma and psychosis is causal; if it is, the size of this association remains uncertain.

Few studies have examined whether different types of trauma affect the risk of psychotic experiences in different ways. Trauma that involves neglect or interpersonal violence appears to be associated with a greater risk of psychotic experiences compared with exposure to unintentional injury, parental loss, or economic adversity.^14,15,16^ However, whether a specific type of interpersonal trauma is more strongly associated with psychosis risk than other types is unclear. In studies that have examined a range of trauma types using multivariable models, sexual abuse has usually been reported to be more strongly associated with psychosis risk than other interpersonal trauma exposures,^6,9,15,17^ although CIs often overlap with those for other types of trauma exposure.^18^

A limited number of studies have examined whether a sensitive or critical period of risk exists during which exposure to trauma is particularly likely to be associated with psychosis. One study reported a stronger association of earlier trauma (before age 7 years) with psychosis but with overlapping CIs for trauma after this age^14^; another study found no evidence of difference for exposure before and after age 13 years,^16^ and yet another^19^ study examined adverse exposures that were differently defined at separate age periods and were thus not directly comparable. Further investigation is, therefore, required to establish whether there are sensitive periods of risk for exposure to maltreatment.

The present study investigated the role of trauma type, developmental age, exposure frequency, and confounding variables in the association between trauma and psychotic experiences. Using data from a well-characterized UK birth cohort, the Avon Longitudinal Study of Parents and Children (ALSPAC), we examined (1) whether a comprehensive measure of trauma exposure, using both child- and parent-reported data during childhood and adolescence, was associated with psychotic experiences at age 18 years and if this exposure was attenuated after adjusting for a comprehensive range of potential confounders or explained by reverse causation, (2) whether evidence existed to support a dose-response association with exposure to multiple types of trauma, (3) whether specific types of trauma were more strongly associated with risk of psychotic experiences than other types, and (4) whether sensitive or critical periods of exposure to trauma existed between 0 and 17 years of age.

## Methods

### Sample

We used data from ALSPAC, a prospective cohort study; the fully searchable data dictionary of ALSPAC is available at http://www.bristol.ac.uk/alspac/researchers/our-data/. The initial cohort consisted of 14 062 children born to women who resided in the former Avon Health Authority area and had an expected delivery date between April 1, 1991, and December 31, 1992. The total sample, including later enrollment phases, comprised 14 775 live births.^20^ Ethical approval for the ALSPAC study was obtained from the ALSPAC Law and Ethics Committee and from local research ethics committees. Participants provided written consent to the collection and use of these data to address research questions approved by ALSPAC. This current study uses fully anonymized ALSPAC data and no clinical or administrative records. Data were analyzed from January 9, 2017, to November 30, 2017.

### Measures

#### Psychotic Experiences

Psychotic experiences were assessed using the psychosis-like symptoms semistructured interview (PLIKSi) at age 12 years and then at age 18 years.^21,22^ The assessment at age 12 years rated psychotic experiences present in the previous 6 months. The assessment at age 18 years rated psychotic experiences occurring since age 12 years (outcome used for primary analyses) and psychotic experiences that were incident in the previous 12 months (outcome used for sensitivity analysis addressing potential reverse causation, whereby the associations between trauma and psychotic experiences might arise from childhood psychotic experiences that lead to trauma). The PLIKSi interviews were carried out by trained psychologists and rated following the Schedules for Clinical Assessment in Neuropsychiatry guidelines.

The PLIKSi questions assessed the presence of 12 psychotic experiences, including hallucinations, delusions, and experiences of thought interference. Psychotic experiences were coded as present if 1 or more experiences were rated as suspected or definitely present (eMethods in the Supplement).

#### Trauma

Trauma variables were derived from the responses to 121 questions about traumatic events in the assessments completed by the parents or self-reported by the participants. Of these 49 assessments, the data from 48 assessments of participants aged 0 to 17 years were reviewed contemporaneously. However, because no self-reported assessment of sexual abuse existed during adolescence and self-reports on emotional neglect and physical abuse at this age were limited, we supplemented the data with information from a questionnaire completed at age 22 years, in which participants were asked about these experiences and the age period during which these had occurred (these data were omitted during sensitivity analyses). The questions used to inform each trauma type (physical abuse, sexual abuse, emotional abuse, emotional neglect, domestic violence, or bullying) and responses regarding the severity and frequency of trauma exposure were carefully selected to ensure that a coding of *exposed* reflected the occurrence that would likely be highly upsetting to anyone who experienced it.

The variables derived represent (1) exposure to any trauma type between ages 0 and 17 years; (2) exposure to any trauma type within a distinct age period: early childhood (0-4.9 years), middle childhood (5-10.9 years), or adolescence (11-17 years); (3) exposure to specific trauma types between ages 0 and 17 years; and (4) exposure to specific trauma types within a distinct age period: early childhood, middle childhood, or adolescence. All trauma variables were coded as binary measures. Variables that reflected the number of trauma types that participants were exposed to during the different age periods were also derived, each ranging from 0 to 6 (eMethods in the Supplement).

#### Confounding Variables

On the basis of the literature in this field, we examined a range of variables as potential confounders, including parental information (psychiatric history, genetic risk for schizophrenia, drug use, criminal history, income, smoking during pregnancy, marital status, and living conditions [all of which were assessed around the participant birth]) and participant information (sex, ethnicity, genetic risk for different mental health disorders, temperament at 6 months, developmental delay at 18 months, and intelligence quotient at 8 years [although this factor could also be a potential mediator of early trauma]). Only confounders that changed unadjusted estimates by 5% or greater were included in the final model (eMethods in the Supplement).

### Statistical Analysis

Data analysis was carried out in Stata, version 14 (StataCorp LLC). Logistic regression was used to calculate odds ratios (ORs) and 95% CIs, and Wald test 2-sided *P* values were used for psychotic experiences associated with exposure to trauma before and after adjusting for confounding. We examined the independent association of specific trauma types by adding all trauma types to the confounder-adjusted model and the dose-response associations by comparing categorical variables modeled as dummy variables with categorical variables modeled as linear terms.

We conducted a series of sensitivity analyses to examine the robustness of our findings. To minimize reverse causation, we examined the association between (1) preadolescent trauma (0-10.9 years) and psychotic experiences by age 18 years in a subgroup of individuals who did not report psychotic experiences at age 12 years and (2) adolescent trauma and past-year incidence of psychotic experiences at age 18 years. To address possible lack of measurement invariance across rater types, we conducted separate analyses of parent-reported and child-reported trauma. To examine the association between trauma and more severe psychotic experiences, we used a narrower outcome of definite psychotic experience and suspected or no psychotic experience at age 18 years. To further examine proximal and distal trauma exposure, we compared the association between trauma in early childhood and psychotic experiences at age 12 years with trauma in middle childhood and psychotic experiences at age 12 years. Finally, to rule out potential recall bias in the measures of trauma that included data from the questionnaire completed at age 22 years, we repeated the sensitivity analyses after omitting those data.

### Study Sample

The complete sample with data on exposure, outcomes, and confounders was 3758 (eFigure in the Supplement). We conducted multiple imputation for the sample that had completed the PLIKSi at age 18 years (n = 4433) by creating 50 imputed data sets (eMethods in the Supplement). Our primary results are presented using the sample with imputed confounder and exposure data (n = 4433). Results of analyses using nonimputed data were similar to those using imputed data (eTables 3, 6, 10, and 12 in the Supplement).

## Results

### Study Sample

As summarized in Table 1, those included in the complete-case analytic sample (n = 3758) compared with those excluded (n = 10 196) were more likely to be female (2111 [56.2%] vs 4636 [45.5%]; OR, 1.54; 95% CI, 1.43-1.67), more likely to come from a higher socioeconomic position (low income: 492 [13.1%] vs 1497 [of 6168 (24.3%)]; OR, 0.38; 95% CI, 0.33-0.43), and less likely to report parental history of drug use or mental health problems (329 [8.8%] vs 978 [of 9669 (10.1%)]; OR, 0.85; 95% CI, 0.75-0.97]). Trauma in early childhood was associated with noncompletion of the PLIKSi at 18 years of age.

**Table 1.** Sample Characteristics of Participants Who Completed the Psychotic Experiences Assessment

Variable	Analytic Sample Availability, No./Total No. (%)		OR (95% CI)	*P* Value
	Included^a^ (n = 3758)	Excluded^b^ (n = 10 196)		
Female sex	2111 (56.2)	4636 (45.5)	1.54 (1.43-1.67)	<.001
Parental drug use	329 (8.8)	978/9669 (10.1)	0.85 (0.75-0.97)	.02
Living condition: ≥1 per room	123 (3.3)	755/9028 (8.4)	0.37 (0.31-0.45)	<.001
Lowest income	492 (13.1)	1497/6168 (24.3)	0.38 (0.33-0.43)	<.001
Maternal educational status: <O levels^c^	639 (17.0)	3084/8640 (35.7)	0.29 (0.26-0.32)	<.001
Parental psychiatric history	617 (16.4)	1781/9365 (19.0)	0.84 (0.76-0.93)	.001

Abbreviation: OR, odds ratio.

^a^ Participants included in the analytic sample were those who had completed the assessment of psychotic experiences at age 18 years.

^b^ The denominators vary for each measure as the number for the participants not included in the analytic sample did not include missing data.

^c^ The O levels are the standard examinations taken by students in the United Kingdom at approximately age 16 years. To have no O levels is a marker of low educational achievement.

The imputed sample of 4433 participants included 2504 (56.5%) females and 1929 (43.5%) males, with a mean (SD) age of 17.8 (0.38) years. Of this sample, 410 participants (9.3%) were rated as having had suspected or definite psychotic experiences at the age-18-year assessment. The frequency of exposure to specific trauma types within each age period was higher in the imputed compared with the complete-case data; 64.5% of the imputed sample reported exposure to trauma between 0 and 17 years of age (eTable 1 in the Supplement). Correlations between trauma types at each age period ranged from 0.01 to 0.72 (eTable 2 in the Supplement). Of the candidate confounding variables examined, sex, parental drug use, living condition, income, and maternal educational status were included in the final adjusted model. Individuals exposed to different types of trauma were, in general, more likely to report more adverse family characteristics, although sex showed differential patterns of association with different trauma types (Table 2).

**Table 2.** Summary Statistics of Confounders and Reported Trauma Exposure Between Age 0 and 17 Years

Trauma Type	Confounding Variable, No. (%)				
	Female Sex	Parental Drug Use	Living in Crowded Condition	Low Income	Maternal Educational Status (<O Levels)^a^
Physical abuse					
Yes	470 (56.3)	86 (10.4)	43 (5.4)	120 (15.9)	158 (19.6)
No	2027 (56.5)	307 (8.6)	118 (3.4)	421 (13.3)	647 (18.6)
Emotional abuse					
Yes	513 (59.2)	109 (12.7)	49 (5.9)	143 (18.0)	163 (19.2)
No	1979 (55.7)	284 (8.1)	110 (3.2)	398 (12.8)	640 (18.6)
Bullying					
Yes	597 (49.0)	102 (8.4)	53 (4.5)	151 (13.8)	242 (20.3)
No	1859 (59.2)	279 (9.0)	102 (3.4)	386 (13.7)	534 (17.5)
Sexual abuse					
Yes	303 (87.1)	33 (9.5)	16 (4.8)	58 (18.3)	166 (48.5)
No	2159 (53.8)	355 (8.9)	136 (3.5)	483 (13.4)	1850 (47.4)
Domestic violence					
Yes	465 (42.7)	123 (15.3)	63 (8.2)	167 (22.9)	167 (21.4)
No	2011 (56.2)	264 (7.4)	93 (2.7)	374 (11.7)	626 (17.9)
Emotional neglect					
Yes	151 (50.0)	28 (9.3)	12 (4.2)	45 (16.5)	57 (19.4)
No	2291 (57.0)	848 (8.7)	141 (3.6)	483 (13.4)	716 (18.3)

^a^ The O levels are the standard examinations taken by students in the United Kingdom at approximately age 16 years. To have no O levels is a marker of low educational achievement.

### Trauma Exposure and Psychotic Experiences

In those with psychotic experiences at age 18 years, 83.8% reported exposure to trauma compared with 62.6% without psychotic experiences (imputed data). Exposure to any trauma experienced up to age 17 years was associated with increased odds of psychotic experiences at age 18 years (OR, 3.13; 95% CI, 2.32-4.22; *P* < .001) (Table 3). Adjusting for confounders attenuated the OR by approximately 10% (adjusted OR, 2.91; 95% CI, 2.15-3.93; *P* < .001). The population-attributable fraction for any trauma experienced up to age 17 years on psychotic experiences at age 18 years was 45% (95% CI, 25%-60%).

**Table 3.** Association Between Exposure to Trauma and Subsequent Psychotic Experiences, by Trauma Type and Exposure Frequency^a^

Trauma Type	% Exposed	Unadjusted		Adjusted^b^		Adjusted^b^^,^^c^	
		OR (95% CI)	*P* Value	OR (95% CI)	*P* Value	OR (95% CI)	*P* Value
Any trauma	64.5	3.13 (2.32-4.22)	<.001	2.91 (2.15-3.93)	<.001	NA	NA
Physical abuse	23.1	2.36 (1.85-3.02)	<.001	1.69 (1.27-2.23)	<.001	2.24 (1.75-2.87)	<.001
Emotional abuse	23.7	1.94 (1.53-2.46)	<.001	1.81 (1.42-2.31)	<.001	1.25 (0.94-1.65)	.13
Bullying	32.9	2.07 (1.66-2.57)	<.001	2.05 (1.65-2.57)	<.001	1.80 (1.43-2.26)	<.001
Sexual abuse	11.0	2.75 (2.00-3.79)	<.001	2.50 (1.79-3.51)	<.001	2.04 (1.42-2.91)	<.001
Domestic violence	21.9	2.02 (1.59-2.56)	<.001	1.79 (1.40-2.29)	<.001	1.48 (1.13-1.94)	.004
Emotional neglect	7.8	2.41 (1.75-3.30)	<.001	1.89 (1.35-2.65)	<.001	2.33 (1.70-3.21)	<.001
No. of trauma types							
1	26.7	1.94 (1.33-2.81)	.001	1.89 (1.30-2.74)	.001	NA	NA
2	16.4	2.67 (1.81-3.91)	<.001	2.54 (1.72-3.75)	<.001	NA	NA
≥3	21.3	5.19 (3.76-7.16)	<.001	4.74 (3.40-6.59)	<.001	NA	NA
NA	Linear trend	1.70 (1.54-1.87)	<.001	1.65 (1.48-1.82)	<.001	NA	NA

Abbreviations: NA, not applicable; OR, odds ratio.

^a^ Imputed data set: n = 4433.

^b^ Adjusted for confounders: sex, low income, parental drug use, maternal educational status, and crowded living condition.

^c^ Adjusted for other trauma exposures.

### Dose-Response Relationship

We observed an increase in effect size with exposure to a greater number of trauma types between age 0 and 17 years (linear trend: adjusted OR, 1.70; 95% CI, 1.54-1.87; *P* < .001) (eTable 4 in the Supplement). Reporting 3 or more types of trauma exposure between age 0 and 17 years was associated with a 4.7-fold increase in odds of psychotic experiences (95% CI, 3.40-6.59; *P* < .001). In addition, evidence shows that exposure to trauma in all 3 age periods was associated with higher risk of developing psychotic experiences than exposure within only 1 or 2 age periods (linear trend: adjusted OR, 1.51; 95% CI, 1.36-1.68) (eTables 5 and 6 in the Supplement).

### Specific Types of Trauma and Psychotic Experiences

Strong evidence supports the association of all trauma types exposed between 0 and 17 years of age with the increased odds of psychotic experiences (adjusted ORs, 1.69-2.50; all *P* < .001) (Table 3). The CIs for associations between specific trauma types and psychotic experiences all overlapped substantially. In the multivariable model adjusting for all trauma types, strong evidence of association with psychotic experiences persisted for physical abuse (adjusted OR, 2.24; 95% CI, 1.75-2.87), sexual abuse (adjusted OR, 2.04; 95% CI, 1.42-2.91), bullying (adjusted OR, 1.80; 95% CI, 1.43-2.26), and emotional neglect (adjusted OR, 2.33; 95% CI, 1.70-3.21). The associations for exposure to domestic violence (adjusted OR, 1.48; 95% CI, 1.13-1.94) and emotional abuse (adjusted OR, 1.25; 95% CI, 0.94-1.65) were substantially attenuated.

### Sensitive or Critical Age Periods of Risk

Exposure to trauma during any of the age periods was associated with increased odds of psychotic experiences (Table 4). Adjusting for confounding had a slightly stronger attenuating effect on the estimate for trauma exposure during early childhood (approximately 20% attenuation) than the estimate for trauma exposure during adolescence (approximately 10% attenuation). Effect sizes were greater for exposure to trauma that was more proximal to the outcome, although the CIs overlapped with more distal exposure.

**Table 4.** Associations Between Exposure to Trauma and Psychotic Experiences at Age 18 Years, by Age Period and Trauma Type^a^

Variable	% Exposed	Unadjusted		Adjusted^b^		Adjusted^b^^,^^c^	
		OR (95% CI)	*P* Value	OR (95% CI)	*P* Value	OR (95% CI)	*P* Value
**Trauma Type (Age Period)**							
Any trauma (0-4.9 y)	22.5	1.88 (1.49-2.38)	<.001	1.70 (1.33-2.17)	<.001	NA	NA
Any trauma (5-10.9 y)	43.6	2.27 (1.81-2.84)	<.001	2.16 (1.71-2.71)	<.001	NA	NA
Any trauma (11-17 y)	40.1	2.92 (2.29-3.71)	<.001	2.72 (2.13-3.47)	<.001	NA	NA
**Trauma Type (0-4.9 y)**							
Physical abuse	4.7	1.32 (0.83-2.09)	.24	1.30 (0.82-2.08)	.26	.93 (0.56-1.55)	.78
Emotional abuse	11.2	1.64 (1.21-2.23)	.002	1.52 (1.11-2.07)	.009	1.31 (0.83-1.86)	.13
Bullying	1.7	1.81 (0.90-3.66)	.10	1.71 (0.84-3.48)	.14	1.68 (0.82-3.43)	.16
Sexual abuse	0.2	3.52 (0.69-17.85)	.13	2.42 (0.46-12.84)	.30	2.47 (0.46-13.26)	.29
Domestic violence	13.2	2.08 (1.60-2.71)	<.001	1.83 (1.39-2.40)	<.001	1.71 (1.27-2.29)	<.001
Emotional neglect	3.5	NA	NA	NA	NA	NA	NA
**Trauma Type (5-10.9 y)**							
Physical abuse	10.3	2.07 (1.52-2.84)	<.001	1.98 (1.45-2.72)	<.001	1.58 (1.10-2.26)	.01
Emotional abuse	12.9	1.86 (1.41-2.45)	<.001	1.77 (1.34-2.35)	<.001	1.37 (0.98-1.91)	.06
Bullying	21.6	1.89 (1.46-2.37)	<.001	1.91 (1.48-2.44)	<.001	1.74 (1.34-2.25)	<.001
Sexual abuse	2.8	1.87 (1.07-3.28)	.03	1.50 (0.84-2.67)	.17	1.18 (0.64-2.17)	.59
Domestic violence	13.1	1.99 (1.46-2.72)	<.001	1.75 (1.26-2.43)	.001	1.47 (1.04-2.08)	.03
Emotional neglect	3.5	2.45 (1.58-3.18)	<.001	2.32 (1.49-3.63)	<.001	1.95 (1.23-3.09)	.004
**Trauma Type (11-17 y)**							
Physical abuse	15.6	2.63 (2.02-3.42)	<.001	2.43 (1.86-3.18)	<.001	1.83 (1.36-2.47)	<.001
Emotional abuse	7.3	2.42 (1.75-3.35)	<.001	2.23 (1.60-3.10)	<.001	1.40 (0.95-2.06)	.09
Bullying	14.4	2.17 (1.69-2.78)	<.001	2.10 (1.64-2.70)	<.001	1.87 (1.45-2.42)	<.001
Sexual abuse	9.4	3.21 (2.31-4.46)	<.001	3.00 (2.12-4.21)	<.001	2.34 (1.62-3.37)	<.001
Domestic violence	5.0	1.99 (1.22-3.23)	.006	1.70 (1.03-2.81)	.04	1.37 (0.80-2.33)	.25
Emotional neglect	3.5	2.33 (1.56-3.74)	<.001	2.29 (1.52-3.44)	<.001	1.96 (1.28-3.00)	.002

Abbreviations: NA, not applicable; OR, odds ratio.

^a^ Imputed data set: n = 4433.

^b^ Adjusted for confounders: sex, low income, parental drug use, maternal educational status, and crowded living condition.

^c^ Adjusted for other trauma exposures.

### Sensitivity Analyses

Results of the association between exposure to both preadolescent and adolescent trauma and subsequent psychotic experiences were substantively the same when excluding participants who reported psychotic experiences at age 12 years (eTable 7 in the Supplement) or when only psychotic experiences at an age-18-year incident within the past year were examined (eTable 8 in the Supplement). Estimations of effect sizes were similar when using a narrower definition of psychotic experiences at age 18 years (eTable 9 in the Supplement) and when comparing effect sizes in middle childhood and adolescence between trauma reported by parents and trauma self-reported by children (eTable 10 in the Supplement). Consistent with the results for proximity of trauma in our primary analyses, exposure to trauma in middle childhood was more strongly associated with psychotic experiences at age 12 years (adjusted OR, 1.80; 95% CI, 1.45-2.16) than exposure to trauma in early childhood (adjusted OR, 1.33; 95% CI, 1.08-1.65), although the CIs overlapped. Finally, when we excluded trauma data collected at age 22 years, the effect sizes were smaller, although the strength of evidence remained similar for most trauma variables (eg, adjusted OR for any trauma at age 0-17 years, 2.62; 95% CI, 2.02-3.41; *P* < .001) (eTable 11 in the Supplement).

## Discussion

Using data from ALSPAC, a large population-based birth cohort, we found that exposure to traumatic experiences during childhood and adolescence was associated with the development of psychotic experiences by early adulthood. This result was not explained by a more comprehensive range of confounders than were adjusted for in any previous study, including genetic risk for psychiatric disorders, family characteristics, socioeconomic adversity, and markers of childhood development. Associations for adolescent trauma were also not explained by reverse causation, providing perhaps the strongest observational evidence to date for the thesis that a causal association exists between trauma and psychotic experiences. That confounding is not an adequate explanation for this association is consistent with findings from other studies.^9,14,23,24^

Exposure to any type of trauma was associated with psychotic experiences, with little evidence that specific types of trauma are associated with an increase in the risk of psychotic experiences more than other types. The risk of psychotic experiences was stronger after exposure to multiple types of trauma or repeated episodes of trauma at multiple age periods, which is consistent with a dose-response relationship and findings from other studies.^25^

Adolescence was the age period during which exposure to trauma was most strongly associated with risk of psychotic experiences. Possible explanations for this pattern of associations include the following: (1) temporal proximity to the outcome affects risk more than age of exposure, and natural resolution of trauma-related psychopathologic status occurs over time, which is consistent with findings from 2 other studies^15,23^; (2) adolescence represents a particularly sensitive period of risk for the association of interpersonal trauma with psychosis, support for which comes from animal and human studies showing an increase in hypothalamic–pituitary–adrenal activation and anxiety after exposure to stress among adolescents compared with other age groups^26,27,28,29^; (3) weaker associations with earlier trauma measures may indicate greater measurement error in our study, perhaps because these measures were obtained from parental reports only, although this explanation seems unlikely given the results from our sensitivity analyses addressing informant-related measurement variance (eResults in the Supplement). Our findings are consistent with another study^19^ but not all studies^14,16,19^ that have examined differential associations of age of trauma exposure with psychotic experiences.

### Possible Mechanisms

Our results are consistent with the thesis that trauma has a causal association with the origin of psychotic experiences and indicate that the mechanism underlying this association is not dependent on the type of trauma but on the severity, chronicity, and perhaps recency of exposure. Biological models of stress show a clear overlap with the dysregulation of dopaminergic and glutamatergic systems,^30^ which are the most widely supported etiological models of psychosis.^31^ Cognitive and perceptual biases that can arise after exposure to trauma,^32^ that are observed more frequently in people with psychosis,^33,34^ and that have been associated with dopaminergic and glutamatergic dysfunction^35^ are strong candidates as mediators of the trauma–psychotic experience relationship; although requiring further evidence,^36^ these mediators might be potential targets for interventions.

### Implications of Findings

This study indicates that, assuming the association is accurate and causal, a substantial proportion (25%-60%, consistent with previous estimates^4^) of participants would not have developed psychotic experiences if they had not been exposed to traumatic experiences during childhood.

Psychotic experiences are associated with the presence of and increased risk of developing a wide range of adverse mental health outcomes apart from psychotic disorders^37,38^ and occur outside of the context of mental illness. Although they may be a nonspecific marker of the severity of general psychopathologic status,^39^ psychotic experiences are associated with substantial levels of distress and impairment at a population-health level.^22^ Novel interventions that aim to address how trauma affects the mechanisms underlying the development of psychotic experiences could improve mental health outcomes in population-based and clinical contexts.

### Strengths and Limitations

This study has several strengths, including its use of a large, population-based birth cohort with multiple measures of trauma collected contemporaneously to minimize measurement error and recall bias; a wealth of relevant data to allow rigorous testing of confounders; and repeated measures of psychotic experiences to minimize reverse causation. Furthermore, we used semistructured interviews (with PLIKSi) to assess psychotic experiences, hence increasing the validity of the outcome and allowing greater confidence in inferring information about the origin of such phenomena.

However, the study also has a number of limitations. First, as with most cohort studies, the study encountered substantial attrition over time, which may have led to selection bias when using complete-case data. We therefore used multiple imputation, with data from a range of relevant variables associated with trauma exposure and with missingness, to make the missing-at-random assumption more plausible and thus minimize potential attrition bias.

Second, most of our exposure data were collected prior to age 18 years, but we lacked data on sexual abuse in adolescence and lacked self-reported measures of physical abuse and emotional neglect during this developmental period. Thus, we obtained this information from an assessment at age 22 years and hence may have been subject to recall bias. Our sensitivity analyses omitted data from this questionnaire, which led to smaller effect sizes in the association between exposure to trauma and psychotic experiences. The smaller effect size could support either the presence of recall bias, leading to an overestimation in the main reported analyses, or greater measurement error, resulting from the loss of any self-reported information on some trauma types during adolescence.

## Conclusions

These findings of consistent associations between different trauma types and psychotic experiences that are not explained by a broad range of confounders or of dose-response relationships and with strongest associations observed for more proximal traumas support the thesis that traumatic experiences could have a causal association with psychotic experiences. These results do not suggest that early childhood is a sensitive age period during which exposure to trauma is particularly likely to be associated with risk of developing psychotic experiences. Longitudinal studies that examine potentially modifiable mediators in the relationship between trauma and psychotic experiences are required to inform prevention strategies and to improve outcomes for a range of mental health disorders.

## Notes

Supplement. **eMethods.** Measurement of Trauma **eResults.** Supplementary Results **eFigure.** Flow Diagram of Included Participants **eTable 1.** Summary Statistics of Trauma Exposure in Complete-Case and Imputed Datasets **eTable 2.** Tetrachoric Correlation Matrix Between Trauma Exposures According to Type and Timing **eTable 3.** Associations Between Exposure to Trauma and Subsequent Psychotic Experiences According to Type and Frequency in Complete-Case Data **eTable 4.** Association Between Exposure to Trauma, by Frequency and Type, and Psychotic Experiences at Age 18 Years **eTable 5.** Associations Between Number of Timepoints Trauma Is Reported and Psychotic Experiences at 18 Years^a^ **eTable 6.** Associations Between Number of Timepoints Trauma Is Reported and Psychotic Experiences at 18 Years Using Complete-Case Data **eTable 7.** Associations Between Trauma Reported Prior to Adolescence and Psychotic Experiences at 18 Years, Excluding Psychotic Experiences at 12 Years **eTable 8.** Associations Between Trauma Reported in Adolescence and Psychotic Experiences Reported at 18 Years Old Incident in Last Year **eTable 9.** Associations Between Trauma and Definite Psychotic Experiences Reported at 18 Years Old^b^ **eTable 10**. Associations Between Trauma Reported by Child or Parent^a^ and Psychotic Experiences Reported at 18 Years Old Exposures Using Complete-Case Data **eTable 11.** Associations Between Trauma Reported and Psychotic Experiences Reported at 18 Years Old Exposures Omitting Data From Age 22 Questionnaire **eTable 12.** Associations Between Exposure to Trauma and Psychotic Experiences at 18 Years According to Number of Trauma Types at Each Age Point Using Complete-Case Data
eReferences
