# Supplementary material for: Association of Trauma Type, Age of Exposure, and Frequency in Childhood and Adolescence With Psychotic Experiences in Early Adulthood
Source: JAMA Psychiatry. 2018 Nov 21;76(1):79–86. doi: 10.1001/jamapsychiatry.2018.3155 (PMC6490231; doi:10.1001/jamapsychiatry.2018.3155)
Supplement: Supplement. — eMethods. Measurement of Trauma eResults. Supplementary Results eFigure. Flow Diagram of Included Participants eTable 1. Summary Statistics of Trauma Exposure in Complete-Case and Imputed Datasets eTable 2. Tetrachoric Correlation Matrix Between Trauma Exposures According to Type and Timing eTable 3. Associations Between Exposure to Trauma and Subsequent Psychotic Experiences According to Type and Frequency in Complete-Case Data eTable 4. Association Between Exposure to Trauma, by Frequency and Type, and Psychotic Experiences at Age 18 Years eTable 5. Associations Between Number of Timepoints Trauma Is Reported and Psychotic Experiences at 18 Yearsa eTable 6. Associations Between Number of Timepoints Trauma Is Reported and Psychotic Experiences at 18 Years Using Complete-Case Data eTable 7. Associations Between Trauma Reported Prior to Adolescence and Psychotic Experiences at 18 Years, Excluding Psychotic Experiences at 12 Years eTable 8. Associations Between Trauma Reported in Adolescence and Psychotic Experiences Reported at 18 Years Old Incident in Last Year eTable 9. Associations Between Trauma and Definite Psychotic Experiences Reported at 18 Years Oldb eTable 10. Associations Between Trauma Reported by Child or Parenta and Psychotic Experiences Reported at 18 Years Old Exposures Using Complete-Case Data eTable 11. Associations Between Trauma Reported and Psychotic Experiences Reported at 18 Years Old Exposures Omitting Data From Age 22 Questionnaire eTable 12. Associations Between Exposure to Trauma and Psychotic Experiences at 18 Years According to Number of Trauma Types at Each Age Point Using Complete-Case Data eReferences [file jamapsychiatry-e183155-s001.pdf]

Supplementary Online Content

Croft J, Heron J, Teufel C, et al. Association of trauma type, age of exposure, and frequency in childhood and adolescence with psychotic experiences in early adulthood. *JAMA Psychiatry.* Published online November 21, 2018. doi: 10.1001/jamapsychiatry.2018.3155

eMethods. Measurement of Trauma

eResults. Supplementary Results

eFigure. Flow Diagram of Included Participants

eTable 1. Summary Statistics of Trauma Exposure in Complete-Case and Imputed Datasets

eTable 2. Tetrachoric Correlation Matrix Between Trauma Exposures According to Type and Timing

eTable 3. Associations Between Exposure to Trauma and Subsequent Psychotic Experiences According to Type and Frequency in Complete-Case Data

eTable 4. Association Between Exposure to Trauma, by Frequency and Type, and Psychotic Experiences at Age 18 Years

eTable 5. Associations Between Number of Timepoints Trauma Is Reported and Psychotic Experiences at 18 Years[a]

eTable 6. Associations Between Number of Timepoints Trauma Is Reported and Psychotic Experiences at 18 Years Using Complete-Case Data

eTable 7. Associations Between Trauma Reported Prior to Adolescence and Psychotic Experiences at 18 Years, Excluding Psychotic Experiences at 12 Years

eTable 8. Associations Between Trauma Reported in Adolescence and Psychotic Experiences Reported at 18 Years Old Incident in Last Year

eTable 9. Associations Between Trauma and Definite Psychotic Experiences Reported at 18 Years Old[b]

eTable 10. Associations Between Trauma Reported by Child or Parent[a] and Psychotic Experiences Reported at 18 Years Old Exposures Using Complete-Case Data

eTable 11. Associations Between Trauma Reported and Psychotic Experiences Reported at 18 Years Old Exposures Omitting Data From Age 22 Questionnaire

eTable 12. Associations Between Exposure to Trauma and Psychotic Experiences at 18 Years According to Number of Trauma Types at Each Age Point Using Complete-Case Data

eReferences

This supplementary material has been provided by the authors to give readers additional information about their work.

© 2019 Croft J et al. *JAMA Psychiatry*.

eMethods. Measurement of Trauma

Selection of Trauma Variables

Questions were selected on the following criteria:

i) how clearly they referred to exposure to a trauma exposure included in the pre-existing trauma categories

ii) The question has a response option that refers to chronic or severe stressors that would be considered traumatic

Selection of questions and responses relating to severity and frequency were carefully considered in the derivation of the variables for use in the study to ensure that these reflected experiences that would likely be highly upsetting to anyone who experienced them. Responses to a range of questionnaires from children and caregivers regarding trauma exposures were used to derive measures of exposure to a range of categorised trauma exposures. Measures were collected prospectively from ages 0 to 17 years old and supplemented by data collected at aged 22 that referred to traumatic exposures that occurred in childhood (before 11) and adolescence (11-17). The number of questions completed by parents and children at each timepoint, and how many were collected retrospectively at age 22 years are reported according to each trauma type. Example questions used to inform the trauma-type categories include: 'In the last year, has someone hit, kicked, punched or attacked you with the intention of really hurting you?' (physical abuse); 'Has an adult or older child forced, or attempted to force, you into sexual activity?' (sexual abuse); 'How often has an adult in the family said hurtful or insulting things to you?' ('often' or 'very often' classified as emotional abuse); 'How often does a caregiver know where you were going, when you went out, in the last year?' ('never' classified as emotional neglect); 'How often have you been threatened or blackmailed?' (more than four times in the last six months classified as bullying); 'Has your partner been physically cruel towards you in the past year?' (asked of parents; domestic violence).

Deriving Measures

All measures were derived as binary measures for each time-point and trauma type. For each type of trauma exposure, a response of 'yes' to any of the included questions was classified as a 'yes' response to a type of trauma. For a response to be classified as 'no', respondents did not respond 'yes' to any of the questions and responded 'no' to a minimum of one question.

Types of Trauma Exposure

Domestic Violence

The measure for domestic violence was derived from questions completed by caregivers and participants. These questions were related to physical acts of violence taking place in the home on a regular basis and would be traumatic for a child to be exposed to. For questions that referred to regular violence occurring between caregivers, any positive response was recorded as an indicator of domestic violence. For questions that referred to a specific instance or act of violence, for example 'has your partner ever physically twisted your arm?', responses that referred to regular occurrences of this, as opposed to a single instance, were recorded as instances of domestic violence.

A total of 11 questions at 0-4.9 8 questions at 5-10.9 and 4 questions at 11-17 all reported by parents were used to derive this measure.

© 2019 Croft J et al. *JAMA Psychiatry*.

Physical Abuse
This measure was derived from questions regarding physical harm to the participant completed by caregivers and the participant regarding harm from caregivers and other adults. Both mothers and their partners were asked whether they or their partner were physically cruel to their children and participants were asked if they had been physically hurt by their parents or another adult. A positive response to any of these questions was recorded as an indicator of physical abuse. An example of this is ‘In the last year, has someone hit, kicked, punched or attacked you with the intention of really hurting you?’, a positive response would be classified as physical abuse.

The following were used to derive the measure: 19 questions from parents at 0-5 years, 13 questions from parents and 5 questions from children at 5-11 years and 5 questions from parents and 11 questions from children at 11-17 years. Of the questions completed by children, 5 questions at 5-10.9 years and 2 questions at 11-17 were reported at 22 years referring to these timepoints retrospectively.

Emotional Abuse
Emotional abuse was derived from questions where parents were asked whether their children had been exposed to emotional cruelty by themselves or their partners. Any positive to response to this question was recorded as an indicator of emotional abuse. Participants were asked whether adults had said hurtful or insulting things to them or if they were threatened with physical harm: responses of ‘often’ and ‘very often’ were recorded as an indicator of emotional abuse.

The following were used to derive the measure: 18 questions by parents at 0-5, 13 questions from parents and 4 questions from children at 5-11 years and 4 questions from parents and 3 questions from children at 11-17 yea**rs.** Of the questions completed by children, 3 questions at 5-10.9 years and 3 questions at 11-17 were reported at 22 years referring to these timepoints retrospectively.

Emotional Neglect
These questions were based on self-report questions relating to how often caregivers take an interest in aspects of the participants’ lives including their whereabouts and what they do in their spare time. Participants that responded ‘never’ to these questions were identified as being emotionally neglected.

There were no questions available for a measure between 0-5 years, 2 questions between 5-11 and 5 questions between 11-17, all completed by children, were used to derive the measure.

Bullying
Questions from caregivers and participants referring to bullying were used as indicators of bullying. For Questions that asked caregivers whether their child was being bullied, the response ‘certainly true’ was recorded as exposure to bullying. We selected questions from a comprehensive assessment of bullying that included a wide range of forms of bullying (i.e name-calling, blackmail, assault). We included bullying that would be the most likely to be highly distressing and traumatic: this included questioned that referred to any form of physical assault and threats of assault or blackmail. In order to be classified as a traumatic exposure, these instances had to occur a minimum four times in the past 6 months.

A total of 2 questions at 0-5 by parents, 3 questions from parents and 4 questions from children at 5-11, 2 questions by parents and 3 questions by children at 11-17.

Sexual Abuse
Caregivers’ reports of whether their child had been exposed to sexual abuse were recorded as indicators of sexual abuse in early life. Any positive response to questions that refer to any adult or older child forcing or attempting to force the participant into sexual activity was recorded as exposure

© 2019 Croft J et al. *JAMA Psychiatry*.

to sexual abuse. Questions that refer to partners pressuring the participants into sexual activity that referred to frequency of this occurring were recorded as indicators of sexual abuse if the participant responded ‘often’ or ‘all the time’.

A total of 3 questions at 0-5 by parents, 4 questions from parents at 5-11, 4 questions from children at 5-11 and 6 questions from children at 11-17. Of the questions completed by children, 2 questions at 5-10.9 years and all 6 questions at 11-17 were reported at 22 years referring to these timepoints retrospectively.

Coding procedure: complete case data

We established criteria to define the minimum number of questionnaires or interviews (a minimum of 50%) that had to be completed by each participant for each trauma-type within each age-period for a response to be coded as ‘trauma absent’ in the complete-case data sample. This was necessary as the large number of assessments combined with levels of missingness meant that restricting our sample to those with data at every assessment was impractical.

A measure of any reported trauma for each of the three age-periods was derived including participants that had responded to the minimum number of questionnaires in each of trauma type categories for that age. Variables referring to trauma across all age-points (0-17 years old) were then subsequently derived using these measures of any trauma type variables for each age point: participants that had a response for each age-point were included as ‘yes’ or ‘no’ in the 0-17 years measure.

When coding the trauma variables without the minimum response criteria, the results of the analysis were substantively the same compared to analysis completed using the above criteria and were very similar to the prevalence in imputed data. The prevalence of any reported trauma between 0-17 was lower when applying the minimum criterion, but estimates of effect were very similar when compared to either the analysis using imputed data or the analysis where trauma variables were derived without the minimum response criteria.

Parent and Child-reported trauma

Only parent-reported data was available for early childhood measures, whilst measures of trauma in adolescence were predominantly child-reported. In categories that used questions from both parents and children in mid-childhood and adolescence (physical abuse, emotional abuse, bullying and sexual abuse in mid-childhood only), children reported a higher level of exposure to trauma in both mid-childhood (21.6% compared to 13.7% by parents) and adolescence (20.21% compared to 7.0% by parents). In these categories the correlation between parent-reported and child-reported trauma was low, ranging from 0.20 to 0.31.

Assessment of Psychotic Experiences at 18 years old

The semi-structured interview (PLIKSi) instrument comprised an introductory set of questions on unusual experiences, and then 12 ‘ core’ questions eliciting key symptoms covering the three main domains of positive psychotic symptoms: hallucinations (visual and auditory); delusions (delusions of being spied on, persecution, thoughts being read, reference, control, grandiose ability and other unspecified delusions); and symptoms of thought interference (thought broadcasting, insertion and withdrawal). For these 12 core items, 7 stem questions were derived from the Diagnostic Interview Schedule for Children–IV (DISC–IV) and 5 stems from sections 17–19 of the Schedules for Clinical Assessment in Neuropsychiatry version 2.0 (SCAN 2.0). Trained psychologists carried out the interviews. Rating following SCAN guidelines, and raters rated down (i.e. suspected rather than definite; none rather than suspected) if unsure. The average kappa value for interrater reliability was 0.72[1]. PEs were coded as present in a binary outcome if one or more of the experiences was rated as “suspected” or “definitely present” vs none.

© 2019 Croft J et al. *JAMA Psychiatry*.

Assessment of Psychotic Experiences at 12 years old

Psychotic experiences reported at approximately 12 years old were collected using the PLIKSi questionnaire according SCAN guidelines and following the same procedure as PEs collected at approximately 18 years old. In the analytical sample, 12.7% (n=428) reported "definite" or "suspected" psychotic experiences at 12 years old. For more information see Horwood and colleagues[2].

Confounding variables

Parental drug use, parental psychiatric history and criminal history questions were derived from self-report measures during pregnancy and when the child was less than six months old; binary measures were used based on 'yes' or 'no' responses to each question. Questions related to drug use when the child was less than six months old and both mothers and their partners and whether they had recently used any illicit drugs including cannabis: any 'yes' response to these questions was classified as history of drug use. Parental psychiatric history was based on whether parents had attempted suicide during pregnancy or since the child was born or if they reported any psychiatric problem that they sought medical help for. Criminal history was based on self-report questions that asked if the parents had encountered trouble with the law during this period. Maternal marital status (married, separated, never married) and maternal education (<O-level, O-level or >O-level) were based on self-report, household income was based on equivalised income reported between 33-47 months of age separated into quintiles. Developmental delay was measured using a score of development based on mother's reported at 18 months using the Denver Developmental Screening Test [3]. Genetic risk using polygenic risk scores derived for bipolar disorder, schizophrenia, major depressive disorder, and neuroticism are used by Jones and colleagues[4], temperament at 6 months assessed by the Carey Infant and Toddler Temperament Scales[5] and IQ at 8 years old assessed using Wechsler Intelligence Scale for Children (3rd edition)[6].

Multiple Imputation

The complete sample with data on exposure, outcomes and confounders was 3,758 (Supplementary figure 1). To address potential bias from attrition, we conducted multiple imputation using the ICE command. 50 imputed datasets were created using information from variables included in our analyses and additional information from 49 variables associated with observed data and missing-ness that would make the missing at random assumption more plausible.
In addition to selected variables described in the 'Confounding Variables' section (income, drug use, sex, crowding living conditions, crime and IQ at 8 years old), the following variables were included in the multiple imputation model: residency (rented or mortgaged), mother's reported alcohol consumption at 6 months old, mother's reported smoking during pregnancy, social class, mother's life events score, borderline personality disorder assessment at 11 years based on the UK Childhood Interview for DSM-IV BPD [7], any ICD-10 or DSM-IV diagnosis at 10 years using the Development Well-Being Assessment (DAWBA; Goodman et al., 2011), symptoms of depression and anxiety measured at 13 years old using the Moods and Feelings Questionnaire (MFQ; [9] self-reported alcohol consumption at 13 years and smoking frequency at 13 years and self-report measures of PLIKS completed at 12, 13, 14 and 16 years old. Predictive mean matching was used for non-normally distributed variables.

© 2019 Croft J et al. *JAMA Psychiatry*.

eResults. Supplementary Results

Child-reported and parent-reported trauma and association with psychotic experiences at age 12 and 18 years

When comparing the strength of the effects between parent-reported and child-reported trauma variables in complete case data in mid-childhood (Parent Reported OR_{adj} 1.65, 95% CI 1.21, 2.25, Child-reported 2.02 95% CI 1.55, 2.64) and adolescence (Parent Reported OR_{adj} 2.27 95% CI 1.55, 3.32). Child-reported (OR_{adj} 2.16, 95% CI 1.66, 2.81) the association with PLIKS18 remained substantial. This was similarly the case in analysing parent-reported and child-reported trauma in mid-childhood and with PEs reported at 12 years old (Child reported OR_{adj} 1.81 95% CI 1.45, 2.26, parent reported OR_{adj} 1.65 95% CI 1.28, 2.14).

eFigure. Flow Diagram of Included Participants

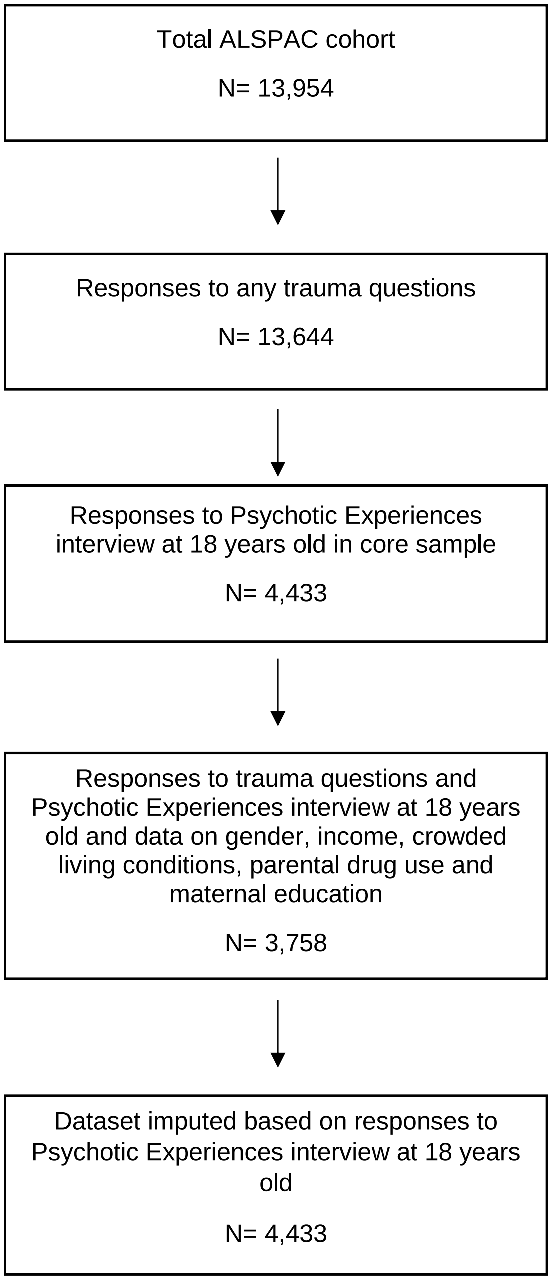

© 2019 Croft J et al. *JAMA Psychiatry*.

eTable 1. Summary Statistics of Trauma Exposure in Complete-Case and Imputed Datasets

	Missing PLIKs18 data %(n)	Frequency of trauma exposure (%)	
		Observed data	Imputed data
Physical Abuse 0-4.9	10.3 (455)	4.2	4.7
Emotional Abuse 0-4.9	10.6 (470)	9.9	11.2
Bullying 0-4.9	11.3 (501)	1.4	1.7
Sexual Abuse 0-4.9	3.7 (162)	.2	.21
Domestic Violence 0-4.9	9.5 (423)	11.6	13.2
Any Reported Trauma 0-4.9	16.8 (744)	16.6	22.4
Physical Abuse 5-10.9	17.6 (782)	8.4	10.3
Emotional Abuse 5-10.9	16.1 (714)	10.8	12.9
Emotional Neglect 5-10.9	9.34 (414)	3.1	3.5
Bullying 5-10.9	11.03 (489)	19.0	21.6
Sexual Abuse 5-10.9	12.7 (557)	2.4	2.8
Domestic Violence 5-10.9	22.9 (1,013)	9.2	13.1
Any reported Trauma 5-10.9	28.8 (1,278)	28.1	43.7
Physical Abuse 11-17	19.0 (842)	12.2	15.6
Emotional Abuse 11-17	24.8 (1,097)	6.5	7.3
Bullying 11-17	4.5 (201)	13.7	14.4
Emotional Neglect 11-17	4.8 (211)	4.0	3.5
Sexual Abuse 11-17	35.5 (1,574)	6.4	9.4
Domestic Violence 11-17	24.8 (1,097)	3.1	5.0
Any reported trauma 11-17	48.8 (2,164)	18.7	38.6
Any reported trauma 0-17	60.0 (2,482)	26.8	64.5

© 2019 Croft J et al. *JAMA Psychiatry*.

eTable 2. Tetrachoric Correlation Matrix Between Trauma Exposures According to Type and Timing

	(1)	(2)	(3)	(4)	(5)	(6)	(7)	(8)	(9)	(10)	(11)	(12)	(13)	(14)	(15)	(16)	(17)	(18)	(19)	(20)
(1) Physical Abuse 0-4.9	1.00																			
(2) Emotional Abuse 0-4.9	0.72	1.00																		
(3) Bullying 0-4.9	0.07	0.02	1.00																	
(4) Sexual Abuse 0-4.9[a]	-	-	-	-	-															
(5) Domestic Violence 04.9	0.50	0.57	0.02	0.17	1.00															
(6) Any Reported Trauma 0-4.9	1.00	1.00	1.00	1.00	1.00	1.00														
(7) Physical Abuse 5-10.9	0.47	0.30	0.04	0.36	0.37	0.36	1.00													
(8) Emotional Abuse 5-10.9	0.56	0.57	0.02	0.14	0.41	0.52	0.61	1.00												
(9) Emotional Neglect 5-10.9	0.14	0.07	0.21	1.00	0.06	0.10	0.15	0.11	1.00											
(10) Bullying 5-10.9	0.11	0.07	0.03	1.00	0.01	0.06	0.22	0.13	0.31	1.00										
(11) Sexual Abuse 5-10.9	0.18	0.24	0.04	1.00	0.04	0.16	0.16	0.23	0.26	0.16	1.00									
(12) Domestic Violence 5-10.9	0.39	0.33	0.05	0.19	0.57	0.48	0.39	0.47	0.13	0.10	0.01	1.00								
(13) Any reported Trauma 5-10.9	0.45	0.36	0.01	0.08	0.39	0.39	1.00	1.00	1.00	1.00	1.00	1.00	1.00							
(14) Physical Abuse 11-17	0.15	0.16	0.04	0.30	0.20	0.22	0.61	0.35	0.24	0.22	0.23	0.19	0.41	1.00						
(15) Emotional Abuse 11-17	0.36	0.33	0.04	0.23	0.26	0.31	0.59	0.61	0.18	0.16	0.25	0.32	0.47	0.59	1.00					
(16) Bullying 11-17	0.17	0.07	0.05	1.00	0.04	0.07	0.20	0.17	0.18	0.26	0.23	0.07	0.23	0.21	0.23	1.00				
(17) Emotional Neglect 11-17	0.05	0.05	0.02	1.00	0.07	0.06	0.04	0.14	0.30	0.07	0.10	0.09	0.10	0.02	0.16	0.24	1.00			
(18) Sexual Abuse 11-17	0.06	0.08	0.04	0.19	0.03	0.04	0.24	0.19	0.18	0.10	0.45	0.01	0.21	0.56	0.32	0.14	0.13	1.00		
(19) Domestic Violence 11-17	0.22	0.17	0.14	-	0.34	0.30	0.27	0.35	0.16	0.04	0.15	0.57	0.32	0.30	0.58	0.12	0.03	0.07	1.00	
(20) Any reported trauma 11-17	0.22	0.17	0.07	0.10	0.20	0.19	0.48	0.35	0.24	0.23	0.31	0.23	0.38	1.00	1.00	1.00	1.00	1.00	1.00	1.00

[a]Note: Dashes in the correlation matrix refer to empty fields in the analysis

© 2018 American Medical Association. All rights reserved.

eTable 3. Associations Between Exposure to Trauma and Subsequent Psychotic Experiences According to Type and Frequency in Complete-Case Data

			Unadjusted			Adjusted[a]			Adjusted[a,b]		
		**N included**	**OR**	**95% CI**	***p***	**OR**	**95% CI**	***p***	**OR**	**95% CI**	***p***
Any Trauma		3,710	2.30	1.77, 2.99	<.001	2.20	1.69, 2.86	<.001			
Physical Abuse		3,588	2.01	1.56, 2.59	<.001	1.98	1.54, 2.56	<.001	1.54	1.12, 2.04	.003
Emotional Abuse			1.67	1.29, 2.16	<.001	1.60	1.24, 2.08	<.001	1.15	.86, 1.55	.340
Bullying			2.08	1.65, 2.63	<.001	2.03	1.61, 2.57	<.001	1.84	1.45, 2.34	<.001
Sexual abuse			1.99	1.41, 2.81	<.001	1.94	1.37, 2.74	<.001	1.53	1.06, 2.21	.022
Domestic Violence			1.71	1.31, 2.23	<.001	1.56	1.19, 2.05	.001	1.32	.99, 1.77	.061
Emotional Neglect			2.18	1.52, 3.11	<.001	2.10	1.47, 3.01	<.001	1.75	1.21, 2.53	.003
Number of trauma types (%)	1 (33.1)<br>2 (18.3)<br>3+ (5.4)	3,758	1.93<br>2.15<br>3.19	1.42, 2.62<br>1.48, 3.12<br>2.34, 4.33	<.001<br><.001<br><.001	1.89<br>2.10<br>3.00	1.39, 2.57<br>1.44, 3.04<br>2.20, 4.10	<.001<br><.001<br><.001			
	Linear Trend		1.44	1.31, 1.58	<.001	1.41	1.28, 1.55	<.001			
**Any trauma (age-period)**											
Any Trauma (0-4.9 years)		3,411	1.62	1.23, 2.11	.001	1.48	1.13, 1.96	.005			
Any Trauma (5-10.9 years)		2,929	2.03	1.54, 2.66	<.001	2.00	1.52, 2.62	<.001			
Any Trauma (11-17 years)		2,064	2.96	2.11, 4.14	<.001	2.84	2.03, 3.99	<.001			

© 2019 Croft J et al. *JAMA Psychiatry*.

Abbreviation: OR, odds ratio [a]Adjusted for confounders: sex, parental income, parental drug use, maternal education, crowded living conditions [b]Adjusted for other trauma exposures

© 2019 Croft J et al. *JAMA Psychiatry*.

eTable 4. Association Between Exposure to Trauma, by Frequency and Type, and Psychotic Experiences at Age 18 Years[a]

Age Period	No. of Trauma Types (% Exposed)	Unadjusted		Adjusted[b]	
		OR (95% CI)	*P* Value	OR (95% CI)	*P* Value
0-4.9 y	1 (15.4)	1.74 (1.31-2.31)	<.001	1.56 (1.79-2.10)	.002
	2 (5.4)	2.27 (1.54-3.36)	<.001	2.03 (1.36-3.02)	<.001
	≥3 (1.5)	1.93 (.93-4.02)	.08	1.82 (.87-3.80)	.11
	Linear trend	1.45 (1.26-1.67)	<.001	1.38 (1.19-1.59)	<.001
5-10.9 y	1 (28.7)	1.80 (1.39-2.34)	<.001	1.75 (1.34-2.28)	<.001
	2 (10.7)	2.80 (2.01-3.91)	<.001	2.65 (1.88-3.73)	<.001
	≥3 (4.3)	4.33 (2.85-6.57)	<.001	3.88 (2.53-5.94)	<.001
	Linear trend	1.65 (1.47-1.85)	<.001	1.60 (1.42-1.80)	<.001
11-17 y	1 (25.9)	2.20 (1.66-2.91)	<.001	2.09 (1.57-2.78)	<.001
	2 (8.9)	3.47 (2.43-4.94)	<.001	3.20 (2.23-4.58)	<.001
	≥3 (3.8)	7.73 (5.12-11.67)	<.001	6.75 (4.42-10.31)	<.001
	Linear trend	1.94 (1.72-2.18)	<.001	1.86 (1.64-2.10)	<.001

Abbreviation: OR, odds ratio.
[a]Imputed data set: n = 4433.
[b]Adjusted for confounders: sex, low income, parental drug use, maternal educational status, and crowded living condition.

© 2019 Croft J et al. *JAMA Psychiatry*.

eTable 5. Associations Between Number of Timepoints Trauma Is Reported and Psychotic Experiences at 18 Years[a]

	**Unadjusted**			**Adjusted[c]**		
**Time-Point Frequency[b] (%)**	**OR**	**95% CI**	***p***	**OR**	**95% CI**	***p***
1 – 27.3	1.54	1.19, 2.00	.001	1.53	1.18, 2.00	.001
2 – 12.9	2.37	1.81, 3.12	<.001	2.29	1.74, 3.02	<.001
3 – 3.88	3.69	2.59, 5.26	<.001	3.45	2.41, 4.95	<.001
Linear Trend	1.54	1.40, 1.71	<.001	1.51	1.36, 1.68	<.001

Abbreviation: OR, odds ratio [a]Imputed dataet (n=4,433) [b]Number of timepoints (early childhood/mid-childhood/adolescence) Trauma exposure is reported [c]Adjusted for confounders: sex, parental income, parental drug use, maternal education, crowded living

© 2019 Croft J et al. *JAMA Psychiatry*.

eTable 6. Associations Between Number of Timepoints Trauma Is Reported and Psychotic Experiences at 18 Years Using Complete-Case Data

		Unadjusted			Adjusted[b]		
**N**	**Trauma Frequency n(%)**	**OR**	**95% CI**	***p***	**OR**	**95% CI**	***p***
3,758[a]	1 – 1,182 (31.4)	1.48	1.13, 1.94	.004	1.48	1.13, 1.94	.004
	2 – 538 (14.3)	2.27	1.67, 3.08	<.001	2.23	1.64, 3.04	<.001
	3 – 107 (2.85)	2.34	1.33, 4.19	.003	2.45	1.37, 4.38	.002
	Linear Trend	1.44	1.26, 1.63	<.001	1.43	1.26, 1.64	<.001

Abbreviation: OR, odds ratio [a]Number of timepoints (early childhood/mid-childhood/adolescence) Trauma exposure is reported [b]Adjusted for confounders: sex, parental income, parental drug use, maternal education, crowded living

© 2019 Croft J et al. *JAMA Psychiatry*.

eTable 7. Associations Between Trauma Reported Prior to Adolescence and Psychotic Experiences at 18 Years, Excluding Psychotic Experiences at 12 Years

	Unadjusted			Adjusted[d]		
**Category**	**OR**	**95% CI**	***p***	**OR**	**95% CI**	***p***
Physical Abuse	1.78	1.25, 2.58	.001	1.80	1.25, 2.60	.002
Emotional Abuse	1.62	1.18, 2.23	.003	1.55	1.12, 2.13	.008
Bullying	1.73	1.28, 2.33	<.001	1.77	1.31, 2.40	<.001
Sexual Abuse	2.00	.99, 3.92	.053	1.59	.78, 3.22	.200
Domestic Violence	2.00	1.47, 2.68	<.001	1.77	1.30, 2.43	<.001
Emotional Neglect	2.00	1.07, 3.76	.031	1.84	.97, 3.47	.062
Any reported trauma	1.90	1.45, 2.48	<.001	1.82	1.38, 2.38	<.001

Abbreviation: OR, odds ratio [a]Trauma Reported between age 0-10.9 years [b]Imputed dataet (n=3,797) [c]Participants who reported definite or suspected psychotic experiences at 12 years old were excluded from analysis [d]Adjusted for confounders: sex, parental income, parental drug use, maternal education, crowded living

© 2019 Croft J et al. *JAMA Psychiatry*.

eTable 8. Associations Between Trauma Reported in Adolescence and Psychotic Experiences Reported at 18 Years Old Incident in Last Year

	Unadjusted			Adjusted[c]				
**Category**	**OR**	**95% CI**	***p***	**OR**	**95% CI**	***p***		
Physical Abuse	2.44	1.47, 3.43	<.001	2.12	1.37, 3.26	.001		
Emotional Abuse	2.56	1.56, 4.25	<.001	2.50	1.50, 4.17	<.001		
Bullying	1.97	1.30, 2.99	.001	1.90	1.25, 2.89	.003		
Sexual Abuse	2.64	1.57, 4.44	<.001	2.51	1.46, 4.32	.001		
Domestic Violence	1.78	.81, 3.83	.149	1.64	.75, 3.61	.218		
Emotional Neglect	1.37	.63, 2.99	.425	1.32	.60, 2.87	.485		
Any reported Trauma 11-17	2.19	1.49, 3.23	<.001	2.08	1.40, 3.08	<.001		

Abbreviation: OR, odds ratio [a]Trauma Reported between age 11 – 16.9 years [b]Imputed dataet (n=4,433) [c]Adjusted for confounders: sex, parental income, parental drug use, maternal education, crowded living [d]Adjusted for exposure to other types of trauma

© 2019 Croft J et al. *JAMA Psychiatry*.

eTable 9. Associations Between Trauma and Definite Psychotic Experiences Reported at 18 Years Old[b]

	**Unadjusted**			**Adjusted[c]**		
	**OR**	**95% CI**	***p***	**OR**	**95% CI**	***p***
Any trauma (0-17 years)	3.06	2.04, 4.60	<.001	2.82	1.87, 4.23	<.001
Any Trauma Early Childhood (0-4.9 years)	1.76	1.29, 2.41	<.001	1.54	1.11, 2.14	.009
Any Trauma Mid-Childhood (5-10.9 years)	2.30	1.70, 3.10	<.001	2.18	1.61, 3.00	<.001
Any Trauma Adolescence (11-17 years)	3.04	2.19, 4.22	<.001	2.79	2.00, 3.90	<.001

Abbreviation: OR, odds ratio [a]Outcome: suspected psychotic experiences classified as 0 in binary measure of psychotic experiences [b]Imputed dataset (n=4,433) [c]Adjusted for confounders: sex, parental income, parental drug use, maternal education, crowded living

© 2019 Croft J et al. *JAMA Psychiatry*.

eTable 10. Associations Between Trauma Reported by Child or Parent[a] and Psychotic Experiences Reported at 18 Years Old Exposures Using Complete-Case Data

			**Unadjusted**			**Adjusted**[b]		
**Time point**		**N**	**OR**	**95% CI**	***p***	**OR**	**95% CI**	***p***
5-10.9 years	Parent-reported	3,615	1.64	1.23, 2.19	.001	1.63	1.22, 2.18	.001
	Child-reported		1.86	1.45, 2.38	<.001	1.86	1.44, 2.39	<.001
11-17 years	Parent-reported	3,512	2.21	1.53, 3.19	<.001	2.18	1.50, 3.18	<.001
	Child-reported		2.24	1.74, 2.87	<.001	2.16	1.68, 2.78	<.001

Abbreviation: OR, odds ratio [a]The following categories included were derived using both parent and child-reported data: 5-10.9 years: physical abuse, emotional abuse, sexual abuse, bullying. 11-17years: physical abuse, emotional abuse, bullying [b]Adjusted for confounders: sex, parental income, parental drug use, maternal education, crowded living

© 2019 Croft J et al. *JAMA Psychiatry*.

eTable 11. Associations Between Trauma Reported and Psychotic Experiences Reported at 18 Years Old Exposures Omitting Data From Age 22 Questionnaire

		**Unadjusted**			**Adjusted**[b]		
**Time point**		**OR**	**95% CI**	***p***	**OR**	**95% CI**	***p***
5-10.9 years	Physical Abuse	1.48	.88, 2.47	.136	1.55	.92, 2.61	.100
	Emotional Abuse	1.70	1.22, 2.36	.002	1.70	1.22, 2.37	.002
	Sexual Abuse	.70	.08, 5.61	.736	.47	.06, 3.78	.476
	Any Reported Trauma	1.55	.91, 2.64	.109	1.74	1.34, 2.27	<.001
11-17 years	Physical Abuse	3.20	2.2, 4.65	<.001	3.02	2.06, 4.46	<.001
	Emotional Abuse	1.83	1.12, 3.01	.017	1.74	1.06, 2.87	.030
	Any Reported Trauma[c]	2.12	1.65, 2.69	<.001	1.96	1.52, 2.52	<.001
0-17 trauma	Any Reported Trauma[c]	2.62	2.02, 3.41	<.001	2.25	1.68, 3.02	<.001

Abbreviation: OR, odds ratio [a]The following categories included were derived using a number retrospective questionnaires at 22 years old and data from these questions are omitted in this analysis: 5-10.9 years: physical abuse, emotional abuse, sexual abuse, 11-17 years: physical abuse, emotional abuse, sexual abuse [b]Adjusted for confounders: sex, parental income, parental drug use, maternal education, crowded living. [c]This measure does not include sexual abuse as the measure for this at this age was derived from the age 22 questionnaire only

© 2019 Croft J et al. *JAMA Psychiatry*.

eTable 12. Associations Between Exposure to Trauma and Psychotic Experiences at 18 Years According to Number of Trauma Types at Each Age Point Using Complete-Case Data

**Time Point**			**Unadjusted**			**Adjusted[a]**		
	**N**	**Trauma Frequency n(%)**	**OR**	**95% CI**	***p***	**OR**	**95% CI**	***p***
0-4.9 years	3,758	1 – 513 (13.7)<br>2 – 190 (5.1)<br>3+ - 53 (1.41)	1.64<br>1.82<br>1.54	1.21, 2.2<br>1.17, 2.83<br>.65, 3.63	.001<br>.008<br>.328	1.50<br>1.66<br>1.51	1.11, 2.04<br>1.06, 2.59<br>.64, 3.59	.008<br>.027<br>.350
		Linear trend	1.33	1.14, 1.56	<.001	1.28	1.09, 1.50	.002
5 – 10.9 years		1 – 972 (26.0)<br>2 – 323 (8.6)<br>3+ - 129 (3.4)	1.57<br>2.00<br>3.14	1.21, 2.04<br>1.39, 2.88<br>1.96, 5.04	.001<br><.001<br><.001	1.57<br>1.96<br>3.07	1.20, 2.04<br>1.35, 2.83<br>1.90, 4.95	.001<br><.001<br><.001
		Linear trend	1.45	1.28, 1.65	<.001	1.44	1.27, 1.64	<.001
11 – 17 years		1 -847 (22.5)<br>2 – 249 (6.6)<br>3+ - 50 (1.33)	1.70<br>3.06<br>8.03	1.30, 2.22<br>2.13, 4.40<br>4.42, 14.6	<.001<br><.001<br><.001	1.67<br>2.94<br>6.94	1.27, 2.19<br>2.04, 4.233<br>3.78, 12.73	<.001<br><.001<br><.001
		Linear trend	1.83	1.60, 2.10	<.001	1.78	1.55, 2.04	<.001

Abbreviation: OR, odds ratio [a]Adjusted for confounders: sex, parental income, parental drug use, maternal education, crowded living conditions

© 2019 Croft J et al. *JAMA Psychiatry*.

eReferences

1. Zammit, S. *et al.* Psychotic experiences and psychotic disorders at age 18 in relation to psychotic experiences at age 12 in a longitudinal population-based cohort study. *Am. J. Psychiatry* **170,** 742–750 (2013).
2. Horwood, J. *et al.* IQ and non-clinical psychotic symptoms in 12-year-olds: results from the ALSPAC birth cohort. *Br. J. Psychiatry* **193,** 185–191 (2008).
3. Iles-Caven, Y., Golding, J., Gregory, S., Emond, A. & Taylor, C. M. Data relating to early child development in the Avon Longitudinal Study of Parents and Children (ALSPAC), their relationship with prenatal blood mercury and stratification by fish consumption. *Data Brief* **9,** 112–122 (2016).
4. Jones, H. J. *et al.* Phenotypic Manifestation of Genetic Risk for Schizophrenia During Adolescence in the General Population. *JAMA Psychiatry* **73,** 221–228 (2016).
5. Sayal, K., Heron, J., Maughan, B., Rowe, R. & Ramchandani, P. Infant temperament and childhood psychiatric disorder: longitudinal study. *Child Care Health Dev.* **40,** 292–297 (2014).
6. Niarchou, M. *et al.* Defective Processing Speed and Nonclinical Psychotic Experiences in Children: Longitudinal Analyses in a Large Birth Cohort. *Am. J. Psychiatry* **170,** 550–557 (2013).
7. Winsper, C., Hall, J., Strauss, V. Y. & Wolke, D. Aetiological pathways to Borderline Personality Disorder symptoms in early adolescence: childhood dysregulated behaviour, maladaptive parenting and bully victimisation. *Borderline Personal. Disord. Emot. Dysregulation* **4,** (2017).
8. Goodman, A., Heiervang, E., Collishaw, S. & Goodman, R. The 'DAWBA bands' as an ordered-categorical measure of child mental health: description and validation in British and Norwegian samples. *Soc. Psychiatry Psychiatr. Epidemiol.* **46,** 521–532 (2011).
9. Siebald, C., Khandaker, G. M., Zammit, S., Lewis, G. & Jones, P. B. Association between childhood psychiatric disorders and psychotic experiences in adolescence: A population-based longitudinal study. *Compr. Psychiatry* **69,** 45–52 (2016).

© 2019 Croft J et al. *JAMA Psychiatry*.
